# First pass vasodilator-stress myocardial perfusion CMR in mice on a whole-body 3Tesla scanner: validation against microspheres

**DOI:** 10.1186/1532-429X-14-S1-P61

**Published:** 2012-02-01

**Authors:** Roy Jogiya, Marcus R Makowski, Alkystis Phinikaridou, Christian Jansen, Niloufar Zarinabad, Amedeo Chiribiri, Rene M Botnar, Eike Nagel, Sebastian Kozerke, Sven Plein

**Affiliations:** 1 Kings College London, London, UK; 2 ETH University, Zurich, Switzerland; 3 LIGHT Institute, Leeds, UK

## Summary

Summary: We report the feasibility of first pass vasodilator-stress myocardial perfusion CMR in mice on a whole-body 3Tesla scanner and demonstrate a 2.6 fold increase of stress over rest myocardial blood flow in good agreement with microspheres.

## Background

Animal models are important to develop our understanding of the pathophysiology of cardiovascular disease and for the development of new therapies. While coronary autoregulation maintains resting myocardial blood flow (MBF) constant over a wide range of pathological conditions, MBF reserve during hyperaemic stress is impaired in several common disease processes. First pass contrast-enhanced myocardial perfusion is the standard CMR method for the estimation of MBF and MBF reserve in man, but is challenging in rodents because of the constraints related to the high temporal and spatial resolution requirements. Murine first pass myocardial perfusion at rest can be performed on a whole-body 3 Tesla CMR system. Hyperaemic myocardial stress perfusion CMR in mice has however not been reported.

## Methods

Five healthy six month old C57BL/6J mice were anaesthetized using 2% isoflurane. CMR imaging was performed on a clinical 3.0 Tesla scanner (Philips Healthcare, Best, the Netherlands) with a 23mm single loop surface coil and a murine monitoring and ECG gating system (SA Instruments, USA). Vasodilator stress was induced using a slow injection of dipyrimadole via a tail vein catheter. Stress perfusion data were acquired with an injection of gadolinium contrast (Gadobutrol 0.5mmol/kg) thirty seconds later. The perfusion pulse sequence has been reported1, in summary, it used a saturation recovery gradient echo method with 10-fold k-space and time domain undersampling with constrained image reconstruction using temporal basis sets (k-t PCA) to achieve a spatial resolution of 0.2 x 0.2 x 1.5mm3 and an acquisition window of 43ms. Following stress perfusion mice were recovered. One week later the mice underwent repeat anaesthesia and stress testing with LV injections of fluorescent microspheres at rest and at stress. Microsphere images were analysed using confocal microscopy.

## Results

**Figure 1:**
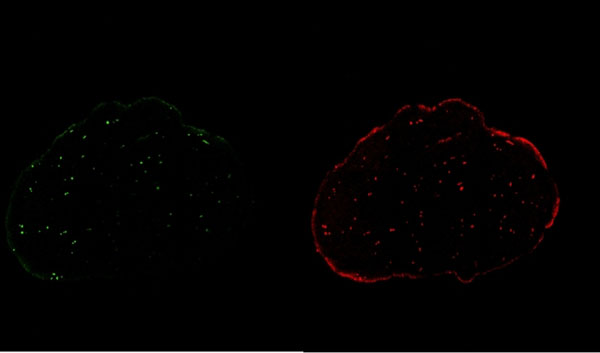
Fluroescent microsphere injections at rest (green) and stress (red).

**Figure 2:**
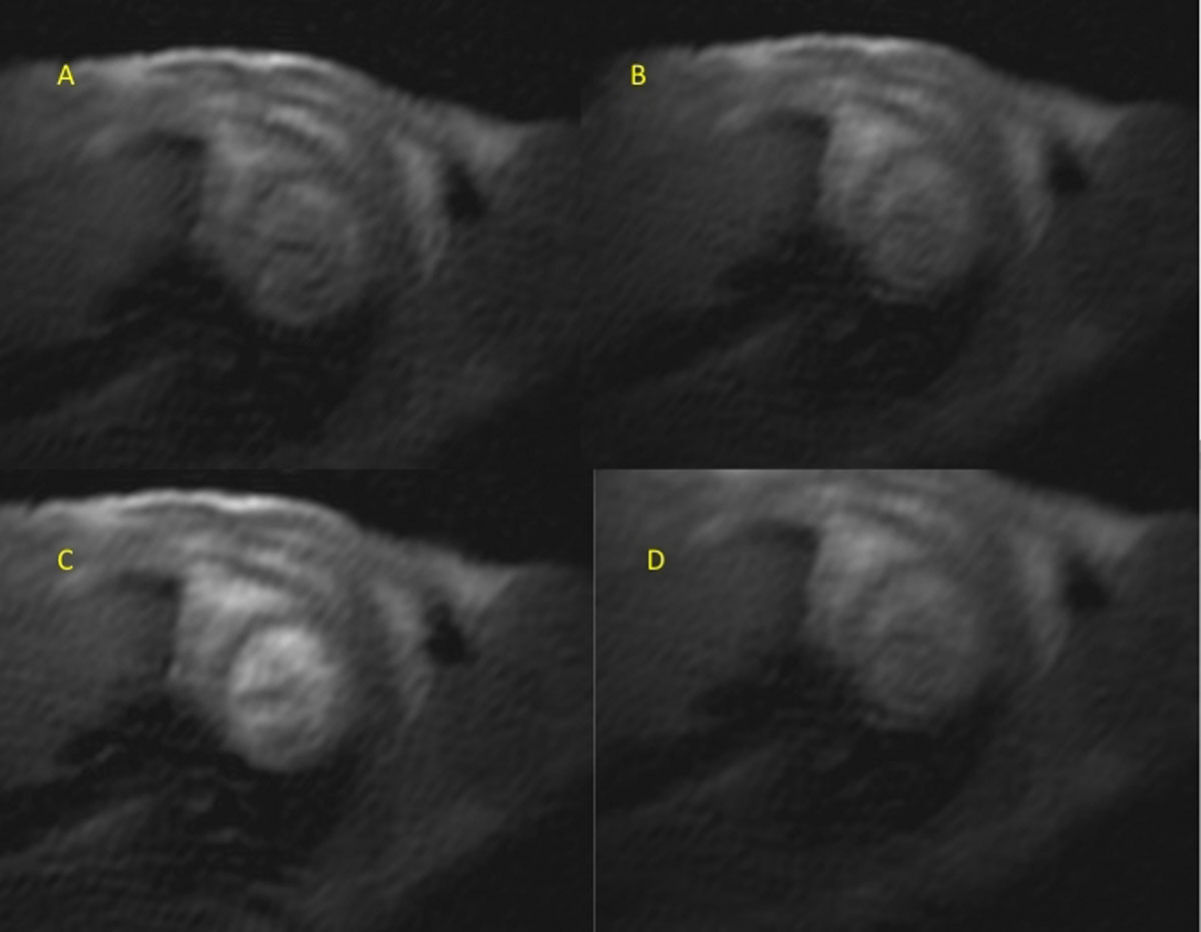
Murine stress perfusion (A)=rest (B)=RV first pass (C)=LV first pass (D)=myocardial wash in.

Data was acquired successfully in all five mice. Mean heart rate increased from 480±27.4 bpm at rest to 503±41.5 bpm (P=0.08) during vasodilatation. Mean myocardial blood flow at rest by Fermi-function constrained deconvolution in control mice was 3.4±0.5ml/g/min and increased to 8.9±3.0ml/g/min during stress (ratio 2.6:1, P=0.036) (Figure 2 a-d). The mean count of microspheres increased from rest to stress by a ratio of 2.7:1 (mean spheres per slice n=27±3.2, n=74±18.5, P=0.0005) (Figure 1).

## Conclusions

First-pass myocardial stress perfusion CMR in a mouse model is feasible. Although the quantification of myocardial blood was lower than published values, the trend in myocardial blood flow was consistent with existing literature. Data was acquired on a 3 Tesla scanner using an approach similar to clinical acquisition protocols, thus facilitating translation of imaging findings between rodent and human studies to elucidate mechanisms and develop therapies for cardiovascular disease.

## Funding

SP is funded by British Heart Foundation fellowship FS/10/62/28409.

SP/EN receives research grant support from Philips Healthcare.
